# The Relationship between Sitting and the Use of Symmetry As a Cue to Figure-Ground Assignment in 6.5-Month-Old Infants

**DOI:** 10.3389/fpsyg.2016.00759

**Published:** 2016-05-31

**Authors:** Shannon Ross-Sheehy, Sammy Perone, Shaun P. Vecera, Lisa M. Oakes

**Affiliations:** ^1^Department of Psychology, University of TennesseeKnoxville, TN, USA; ^2^Institute of Child Development, University of MinnesotaMinneapolis, MN, USA; ^3^Department of Psychology, The University of IowaIowa City, IA, USA; ^4^Department of Psychology, University of California, DavisDavis, CA, USA

**Keywords:** infant perception, figure-ground segregation, sitting ability, symmetry, motor development, perception for action

## Abstract

Two experiments examined the relationship between emerging sitting ability and sensitivity to symmetry as a cue to figure-ground (FG) assignment in 6.5-month-old infants (*N* = 80). In each experiment, infants who could sit unassisted (as indicated by parental report in Experiment 1 and by an in-lab assessment in Experiment 2) exhibited sensitivity to symmetry as a cue to FG assignment, whereas non-sitting infants did not. Experiment 2 further revealed that sensitivity to this cue is not related to general cognitive abilities as indexed using a non-related visual habituation task. Results demonstrate an important relationship between motor development and visual perception and further suggest that the achievement of important motor milestones such as stable sitting may be related to qualitative changes in sensitivity to monocular depth assignment cues such as symmetry.

## Introduction

Since the 1960s, we have made considerable advances in our understanding of perceptual development. Research has documented the timing of developmental milestones in infants' perception of color (Dannemiller and Hanko, 1987; Mercer et al., 2014) and depth (Yonas et al., 2002; Hirshkowitz and Wilcox, 2013; Adolph et al., 2014), their ability to segregate objects and detect object boundaries (Cohen and Cashon, 2001; Needham, 2001; Hayden et al., 2011), discriminate between symmetrical and asymmetrical figures (Fisher et al., 1981) as well as factors that influence perceptual and information processing (e.g., Banks and Ginsburg, 1985). This research has led to new insights regarding the interaction between the developmental state of the motor system, and opportunities to detect and learn about the features of objects (e.g., Gibson, 1988; Bushnell and Boudreau, 1993). For example, infants' activity with objects is related to their ability to attend to the appearance of objects embedded in dynamic events (Perone et al., 2008), 3-D object completion is related to infants' manual exploration of objects (Soska et al., 2010), perception of self-propelled motion is related to crawling (Cicchino and Rakison, 2008), and object exploration—including the level of visual attention to objects—is related to infants' experiences picking up objects (Needham et al., 2002). The goal of the present investigation is to extend this literature by examining the relation between independent sitting and figure-ground (FG) segregation in 6.5-month-old infants.

Figure-ground (FG) assignment is vital to visual perception—it allows us to extract the meaningful objects (i.e., figures) from the less meaningful spaces between objects (i.e., grounds). Consequently, faulty coding of figural status could have a catastrophic effect on which items were tagged for further visual and cognitive processing. Because foreground figures occlude other objects, figures are likely to be perceived as close objects in the visual environment. Therefore, FG assignment is intimately tied to depth perception (Palmer, 1999; Vecera et al., 2002). Understanding FG assignment has been of interest to psychologists for decades, and a large body of research has shown that adults use a variety of Gestalt cues, such as symmetry (e.g., Baylis and Driver, 1995), convexity (Kaniza and Gerbino, 1976), and lower region (Vecera et al., 2002) in FG assignment.

Perhaps even more than adults, it is critical that infants solve the problem of FG segregation to guide visual attention, eye movements, and learning. At present, however, we know relatively little about infants' developing sensitivity to Gestalt cues to FG assignment. Segregating visual displays into figure and ground is related to the assignment of visual regions to different depth planes, or *near* vs. *distant* objects (see Palmer, 1999). These depth assignments are important, as they directly influence both infant attention, and perception-for-action cognitions such as when planning a reach. Research has shown that in both looking and reaching tasks, infants prefer the nearer of two objects (Granrud et al., 1984; Craton and Yonas, 1988). During the first postnatal 6 months, infants appear to rely on motion cues to determine what objects are close. For example, von Hofsten and Spelke (1985) observed that when shown a display in which a smaller, closer object occluded a larger, more distant object, 5-month-old infants reached for the closer of two objects in the display, only when the near object moved independently in front of the distant object. Infants did not reach more for the near object when the two objects remained static or when they moved in unison. Similarly, Craton and Yonas (1988) reported that 5-month-old infants reached to a field of dots that moved as if they occluded another field of dots, suggesting they used apparent “boundary flow” to assign depth. Such findings demonstrate that the ability to use motion as a cue to FG assignment develops relatively early, and may rely on the development of visual abilities such as smooth pursuit eye movements and the ocular following response (Nawrot and Nawrot, 2013).

Between 5 and 7 months of age, infants become increasingly sensitive to monocular (pictorial) depth cues (e.g., texture, interposition, and size) in static displays (Walk and Dodge, 1962; Sen et al., 2001; Yonas et al., 2002; Kavsek et al., 2009). For example, 7-month-old infants, but not 5-month-old infants, consistently reach for the “closer” region as specified by pictorial depth cues (Yonas et al., 2002), though a more recent meta-analysis of 16 preferential reaching studies suggests even 5-month-old infants may use pictorial depth cues for depth assignment when viewing the displays monocularly, though the effects are smaller (Kavsek et al., 2009). These findings suggest that infants are able to use pictorial depth cues such as shape, size, and interposition to make judgments about which objects are in front of other objects, a fundamental component of FG assignment, and that this ability develops sometime between 5 and 7 months.

In the present investigation we asked whether sensitivity to the Gestalt cue of symmetry, a robust FG segregation cue in adults (Bahnsen, 1928; see also Palmer, 1999; 2002), also emerges during this age range. By 4 months of age, vertical symmetry enhances encoding and memory (Bornstein et al., 1981; Fisher et al., 1981), however, it is unclear if this early processing advantage is sufficient to drive FG segregation when the symmetrical region is presented as part of a complex object. Given previous findings of relations between motor development and visual perception of objects in this age range (Perone et al., 2008; Soska et al., 2010; Baumgartner and Oakes, 2013), we hypothesized a relation between motor development and sensitivity to symmetry as a cue to FG segregation.

A major motor achievement during this time is the ability to sit upright, without support from the arms. This is a critically important motor achievement that has a cascading effect on infants' perception of many aspects of the visual world. For example, self-sitting creates new opportunities for reaching and manual exploration, which in turn can shape how infants' visually perceive objects. When infants sit independently, both arms are free to extend away from the body, facilitating reaching, and haptic exploration (Adolph and Berger, 2005), and stable reaching has been shown to be preceded by the ability to sit unsupported (Spencer et al., 2000). Therefore, infants who can sit without support should demonstrate increased visual attention toward displays that contain “reachable” targets, or targets that invoke a strong percept of *objectness*. Two findings in the literature support this idea. First, Corbetta et al. (2014) demonstrated infants with more reaching experience devoted more pre-reach visual attention to the *graspable* portion of the target (e.g., the handle), suggesting that reaching experience shaped how infants visually regarded objects prior to and during the act of reaching. In a very different context, Soska et al. (2010) found that the amount of time infants spent looking at objects while they manipulated them was associated with their 3-D completion of a completely different set of visually presented objects. Because looking while manipulating objects is made possible by self-sitting, Soska et al.'s finding further supported our hypothesis of the cascading effect of self-sitting on infants' visual perception of objects; specifically, sitting independently allows infants to explore more with their hands and reach for objects in their environment, thus providing opportunities to learn new properties of objects and visual statistical regularities that indicate object properties.

Another possible cascading effect of sitting is that infants who self-sit spend a greater proportion of their time in a vertical position. This new visual perspective may heighten infants' attention to the statistical regularity of vertical symmetry that defines faces and numerous objects in their environment. Consistent with this possibility, work with sighted, and early blind adults (blind at or near birth) suggests that sensitivity to vertical symmetry is learned though visual experience. Specifically, sighted but blindfolded adults were able to haptically reconstruct configurations that were vertically symmetrical significantly better than both horizontal and asymmetrical configurations. In contrast, early blind subjects showed no special benefit of vertical symmetry (Cattaneo et al., 2010). Moreover, late blind subjects (blind 5 years of age or older) performed no differently than sighted subjects, further supporting the link between early visual experience and sensitivity to the special properties of vertical symmetry (Cattaneo et al., 2013). These results suggest that visual, rather than haptic experience drives sensitivity to vertical symmetry (Cattaneo et al., 2010, 2013).

Here we provide a further test of our hypothesis that self-sitting supports increasing sensitivity to objects defined by vertical symmetry, by asking whether infants who sit independently are more sensitive to *symmetry* for FG assignment. In displays that contain both a symmetrical region and an asymmetrical region, adults tend to perceive the symmetric region as figure and assign the shared edge to that region (Bahnsen, 1928; Baylis and Driver, 1995), presumably reflecting the recognition that the likelihood of any two edges accidentally forming a symmetrical shape is exceedingly rare. We know from previous work that infants are sensitive to symmetry in visual displays (Bornstein et al., 1981; Fisher et al., 1981). We do not yet know when they recognize symmetry as a cue to figure-ground assignment.

**Figure 1:**
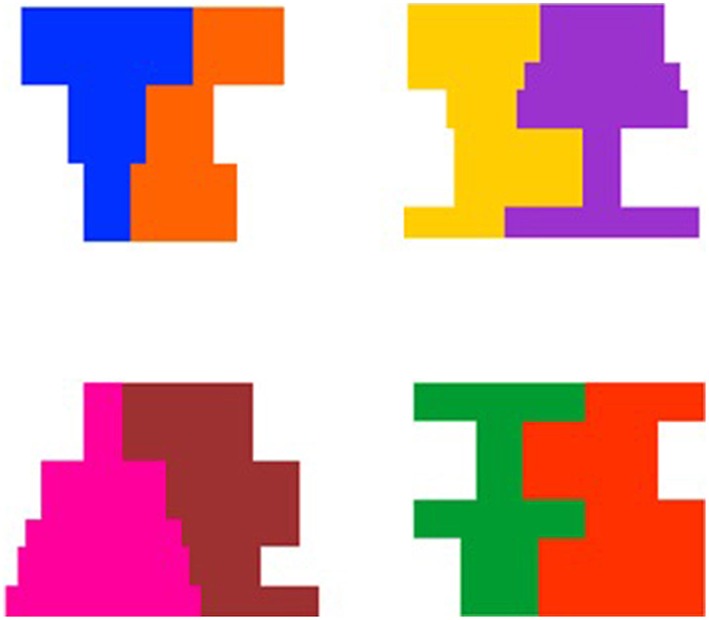
**Examples of the 4 shape composites and 4 color pairs used in Experiments 1 and 2**.

We examined FG segregation using a version of the preferential looking technique (e.g., Fantz, 1958; Ross-Sheehy et al., 2003). On each trial, we presented infants with two identical visual events, each of which was composed of a abutting symmetrical and asymmetrical regions (see Figure 1). After a 2 s delay, one of the regions in each composite moved, producing a percept of either the symmetrical region as figure, or the asymmetrical region a figure. Thus, there are two possible cues for FG assignment, the shape of each region (symmetrical or asymmetrical) and the motion-defined figure, and these cues could either be consistent (both reveal symmetrical region to be figure) or inconsistent (motion reveals asymmetrical region to be figure). To control for the possibility that infants may simply attend to the moving segment, we created two different types of moving events, move in front events (either symmetrical or asymmetrical) or move behind events (either symmetrical or asymmetrical). Events in which that symmetrical region moves in front of the asymmetrical region (e.g., occluding and unoccluding the asymmetrical region) or in which the asymmetrical region moves behind the symmetrical region (e.g., becoming occluded and unoccluded by the symmetrical regions) contain *consistent* FG assignment cues. Events in which the symmetrical region moves behind the asymmetrical region or in which the asymmetrical region moves in front of the symmetrical region are *inconsistent* FG assignment cues.

A significant preference for consistent events would indicate that infants perceive the shared contour as belonging to the symmetric region. This edge assignment is a necessary requirement for perceiving the symmetric region as figure, as a shared contour is assigned to the nearer, occluding object (i.e., the figure) (Driver and Baylis, 1996) and may be critically involved in object based attention (von der Heydt, 2015). Given that infants as young as 5 months use motion as a cue to depth (Craton and Yonas, 1988), we expected that infants who are not sensitive to symmetry as a cue to FG assignment should see a motion-defined figure on each display and show no systematic preference—that is, these infants would use motion alone to assign the shared contour, and would not find it inconsistent if the object in front (the figure) was asymmetrical. In Experiment 1, we assessed the ability to sit unsupported via parental report. In Experiment 2, we assessed the ability to sit unsupported via an in-laboratory sitting assessment, and we assessed infants' habituation in an unrelated task to address the possibility that sitting and non-sitting infants differed in other ways aside from their perception of symmetry as a figure-ground cue.

## Experiment 1

### Method

#### Participants

The final sample included 36 6.5-month-old infants. We divided infants into *sitters* and *non-sitters* based on parental report. Specifically, an experimenter verbally asked parents “Can your infant sit unsupported.” Although this is a coarse measurement of sitting, any differences we observe between these two groups of infants provides a first insight into whether sitting ability is related to FG segregation (Note: Experiment 2 included an in-lab assessment of sitting to validate parental report). In Experiment 1, 18 infants (*M* = 27.54 weeks, *SD* = 0.99 weeks, range = 26.0–29.57 weeks, 11 girls and 7 boys) were reported to be sitters, and 18 infants (*M* = 28.4 weeks, *SD* = 1.34 weeks, range = 25.14–30 weeks, 9 girls and 9 boys) were reported to be non-sitters. We tested an additional 9 infants, but excluded their data from the final analyses due to fussiness (*n* = 4), equipment failure (*n* = 3), experimenter error (*n* = 1), or parental interference (*n* = 1).

Infants were from predominantly middle-class families and were reported to be Caucasian (*n* = 30), African American (*n* = 2), Asian (*n* = 2), or Hispanic (no race reported) (*n* = 2). All infants were healthy and full-term, with no birth complications or vision problems. All mothers had graduated from high school, and 80.48% had completed at least a bachelor's degree. In each experiment reported here, infants' names were obtained from county birth records. When infants approached 6 months of age, parents were contacted by letter, and received a follow-up phone call to schedule an appointment. Parents were reimbursed for parking and infants received a small toy for their participation.

#### Stimuli

**Figure 2:**
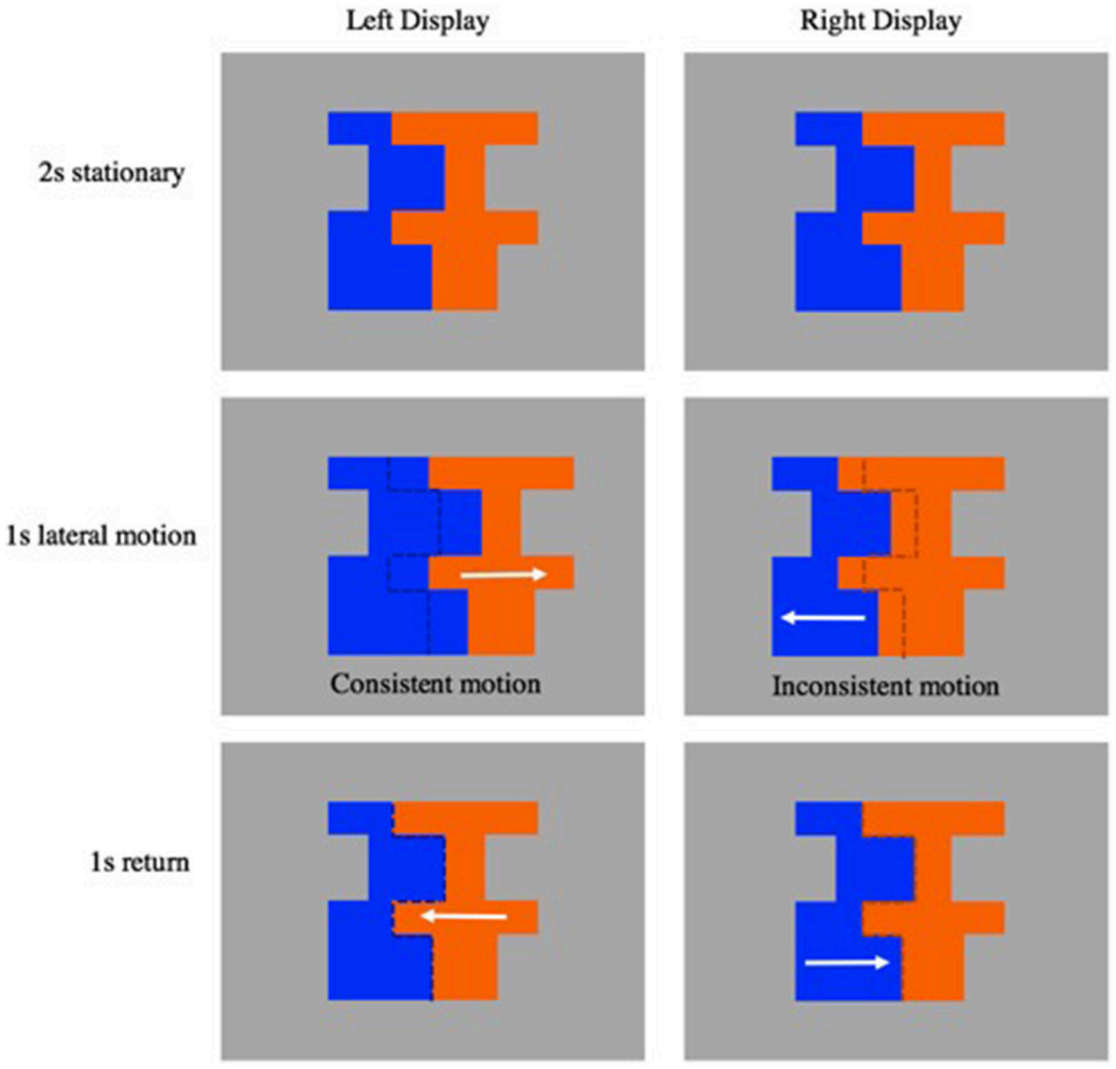
**Depicts a single matched motion trial**. The left display depicts a move in front stimulus event with consistent symmetry and motion cues. The right display depicts a move in front stimulus event with inconsistent symmetry and motion cues. White arrows indicate the direction of segment motion.

Stimuli were computer-generated events that involved a composite composed of adjacent symmetrical and asymmetrical regions (see Figure 1). There were four composites in which the symmetrical and asymmetrical region differed in shape but were matched on overall area ranging in size from 11.2 cm (w) by 10.6 cm (h) to 14.2 cm (w) by 10.6 cm (h), and subtending a visual angle of 6.39° by 6.05° to 8.08° by 6.05° at a viewing distance of 100 cm. In addition, for half of the events involving each composite, the symmetrical region was on the left of the display and for the other half of the events the symmetrical region was on the right of the display. An example of each composite shape is presented in Figure 2.

We used these composites to create several stimulus events. In each stimulus event, the composite was presented as a static image for a period of 2 s, allowing the infant to make an initial FG judgment. Next, either the symmetrical or the asymmetrical segment slid laterally away from the other segment (duration of 1 s and a distance of 1.2–2.2 cm depending on composite shape), then returned to its original position (duration 1 s). The entire sequence took 4 s, and was repeated 5 times to create each 20 s trial. The events involved either *move-in-front* or *move-behind* movement. When one segment moved *in front of* another region it partially occluded the other segment; as a result, the shared contour is assigned to the *moving* region (i.e., the nonmoving segment appeared to be unoccluded and occluded as the moving segment moved back and forth). When one segment *moved behind* the other segment, it became partially occluded by the other region; as a result, the shared contour is assigned to the *nonmoving* segment (i.e., the moving segment appeared to be unoccluded and occluded as it moved back and forth). Importantly, this edge assignment happens automatically, producing a robust perception of the front occluding region as figure in both cases (see Figure 2; Supplementary Videos 1–4).

Five factors (composite shape, color pair, left/right location of symmetrical region, symmetry of moving region, and movement type) were crossed to create 128 stimulus events. Half of the resulting events were *consistent* with respect to symmetry and motion cues—both shape and motion revealed the symmetrical region to be the figure, the other have were *inconsistent* with respect to symmetry and motion cues (see Figure 2 for examples of the consistent and inconsistent events).

We created 12 pseudo-random orders each consisting of 8 paired-comparison trials (each including a consistent and an inconsistent event), with the following constraints: First, within each order, there were trials with every possible color pair counterbalanced for left/right position—i.e., all infants saw blue/orange, orange/blue, yellow/purple, purple/yellow, pink/brown, brown/pink, green/red, red/green. The order in which these color pairs were presented was determined randomly in each order. Second, within each order, there were trials with every possible composite shape, also counterbalanced for left/right presentation, again presented in a random order. Each of the 8 paired comparison trials was created by combining two identical composites (shape and color), one whose segment motion and motion type produced an inconsistent event, and the other whose segment motion and motion type produced a consistent event. Thus, across the 8 trials, each infant saw each composite shape, each color pair, and every possible combination of movement type, and segment motion, with the constraint that one always be consistent, and the other inconsistent. Left/right location of the consistent event was random.

#### Apparatus

Infants were tested in a dimly lit room. A black curtain hung from the ceiling to the floor to divide the room. There were four openings in the curtain. Two of the openings revealed two 17″ (43.2 cm) ViewSonic CRT monitors, one opening in between the computer monitors revealed a small, black box that blinked and produced a beeping sound at a rate of 3 Hz to orient infant attention toward the computer monitors, and one opening revealed a small, low light TV camera lens. Stimuli were presented via a Macintosh G4 computer using software developed for the Macintosh (Cohen et al., 2000–2002).

#### Design

On each trial, infants were shown two events, side-by-side. The same composite (i.e., same shape and color) was presented in each event, but one event was a *consistent event* and the other was an *inconsistent event*. Thus, during the initial 2 s period when the composites were stationary, the two events were identical. Only when the regions began to move did it become apparent which event was consistent, and which was inconsistent.

The 8 trials were divided based on *motion alignability*. Specifically, we reasoned that infants would be better able to compare the two types of events if the motion was aligned, or both involved the same type of motion (Gentner et al., 2007). Therefore, for each infant, half of the trials involved *matched* motion, such that both events involved the same kind of movement (e.g., both contained move behind events or move in front events, see for example Figure 2). The other trials involved *unmatched* motion, such that each event incorporated a different type of movement (e.g., *one* event contained a move behind event, while the other contained a move in front event). Because we reasoned that it would be easier to process the differences between the events when the motion was aligned, we included this factor in our analyses.

#### Procedure

A paired-comparison procedure was used to assess infants' sensitivity to symmetry as a cue to FG segregation. To accomplish this, infants were seated on their parent's lap 100 cm in front of the two monitors. Parents wore opaque glasses to prevent bias. Infants received 8 20-s trials. On each trial, a consistent event was presented on one monitor and an inconsistent event was presented on the other monitor, left and right position of the consistent event counterbalanced across trials. As mentioned above, half of the trials were matched motion trials and half were unmatched motion trials

A trained observer sat out of sight and watched the infant on a video monitor connected to a low-light video camera. At the beginning of the experiment and between each subsequent trial, a beeping, flashing light was used to attract the infant's attention to a location between the two monitors. When the observer determined that the infant was looking at this attention-getter, he or she pressed a computer key, simultaneously ending the attention-getter, and beginning the stimulus presentation. The observer was unaware of which stimulus was being presented, and of the infant's sitting status. Looking to the right and left monitors was recorded on-line by pressing and holding one computer key when the infant was looking to the left and another computer key when the infant was looking to the right. Infants were free to look at either or both monitors during each trial for up to 20 s. If no looking was observed in the first 10 s the trial was stopped, and repeated. A different trained observer also recorded the looking times for 25% of the infants off-line from a video record of the session. Mean inter-observer correlation for the duration of looking on each trial was high, *r* = 0.98, and the mean absolute difference between observers for the duration of looking was low, *M* = 0.46 s. Only data from the primary observer are reported here.

### Results

**Figure 3:**
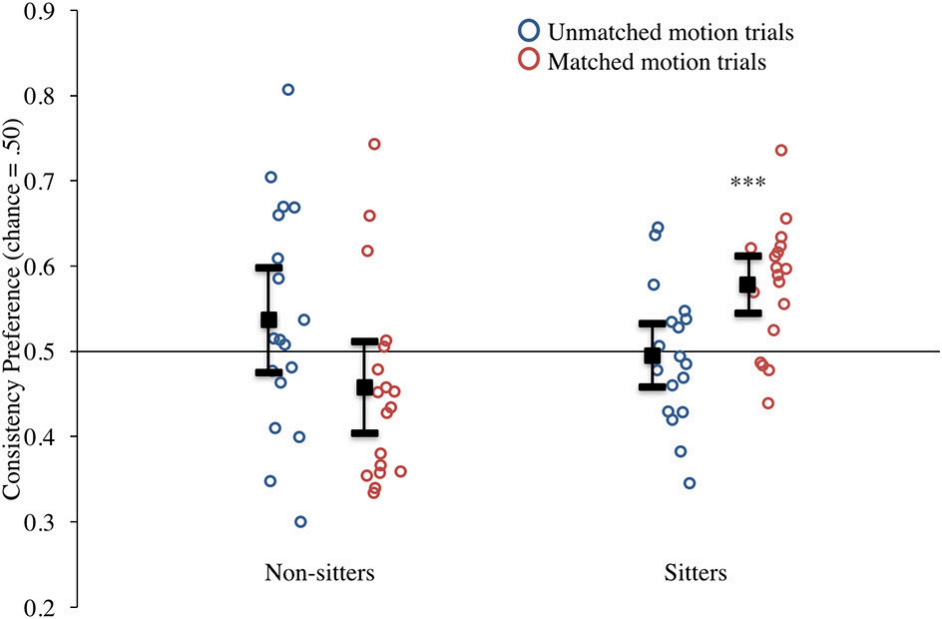
**Experiment 1 consistency preference scores for non-matched and matched motion trials by sitting status**. Circles represent individual infants, solid black square represents mean responding. (Note: ^***^denotes *p* < 0.001, error bars represent 95% confidence intervals).

Infants' preferences for the consistent event were calculated by dividing the duration of their looking to the consistent event by the total amount of looking to both events combined. These preferences are presented in Figure 3. Looking first at results from sitting infants (right half of the figure), it can be seen that the majority of infants who could sit independently showed a preference for the consistent event that was greater than chance (0.50) on *matched* motion trials (red circles) but not on *unmatched* motion trials (blue circles). This impression was confirmed by a series of *t*-tests comparing these preference scores to chance (0.50). Infants whose parents reported they could sit unsupported had a strong and significant preference for the consistent event in on matched motion trials, *t*_(17)_ = 4.50, *p* < 0.001, *d* = 2.18, but not on unmatched motion trials, *t*_(17)_ = −0.28, *p* = 0.79, *d* = −0.13 (see Figure 3). One possible reason that infants exhibit a preference in the matched motion trials is because the motion is alignable, which may facilitate comparison.

Infants whose parents reported that they could not yet sit unsupported are presented in the left half of the figure. In contrast to the independent sitters, these non-sitters showed no systematic preferences in either type of event, and showed increased variability for both matched and unmatched motion trials (see Figure 3). Comparisons to chance again confirmed our initial impressions. These infants' mean preference for the consistent event was not significantly different from chance for either the matched motion trials, *t*_(17)_ = −1.55, *p* = 0.14, *d* = −0.75, or unmatched motion trials, *t*_(17)_ = 1.16, *p* = 0.26, *d* = 0.56. Therefore, we have no evidence that 6.5-month-old infants whose parents report that they do not yet sit unsupported are sensitive to symmetry as a cue to FG assignment.

Further, the graph suggests that sitters and non-sitters responded differently to these two types of trials, a conclusion that was confirmed with an ANOVA conducted on the mean consistency preference scores with sitting status (sitters vs. non-sitters) as the between-subjects variable and condition (matched vs. unmatched motion) as the within-subjects variable. This analysis revealed only a significant sitting status by condition interaction, *F*_(1, 34)_ = 10.76, *p* = 0.002, η^2^ = 0.240. We conducted *post-hoc* analyses using two tailed *t*-tests with a Bonferroni's correction for multiple comparisons (and thus only comparisons with *p* ≤ 0.0125 were considered significant). These comparisons revealed that the preference scores of sitters and non-sitters did not differ significantly for the unmatched motion trials, *t*_(34)_ = 1.14, *p* = 0.26, *d* = 0.39, but the preference scores for the sitters were significantly greater than the non-sitters for the matched motion trials, *t*_(34)_ = −3.72, *p* = 0.001, *d* = 1.28. Similarly, when comparing within each sitting group, we found that sitters showed significantly greater consistency preference scores for matched motion trials than non-matched motion trials, *t*_(17)_ = −2.86, *p* = 0.01, *d* = −1.39, whereas the difference between matched and non-matched motion trials for non-sitters was not significant, *t*_(17)_ = 1.98, *p* = 0.06, *d* = 0.96 ^1^.

### Discussion

These results show that infants whose parents reported that they could sit unsupported can use symmetry as a cue to FG segregation. One limitation of this study was that we relied on parental report to determine sitting status, we are dependent on parental impression of their infants' sitting ability. Although Rochat and Goubet (1995) reported 100% agreement between sitting status obtained via an in-laboratory sitting assessment and parental report, parents may not accurately report their own infant's sitting ability, either due to differences in willingness to call sitting “stable” or some other factor. To address this limitation, in Experiment 2 we conducted an independent in-laboratory sitting assessment, allowing us an unbiased assessment of both overall sitting status as well as stable sitting.

A second potential limitation of Experiment 1 is that although we observed a connection between independent sitting and FG segregation, it is impossible to know whether this relation is unique, or whether independent sitting is correlated with cognitive development more generally. To rule out the possibility that sitting infants are simply more developmentally mature, compliant or visually attentive than non-sitting infants, in Experiment 2 we additionally assessed infants' ability on a separate task of processing speed and memory.

## Experiment 2

### Method

#### Participants

The final sample consisted of 44 6.5-month-old infants divided into sitters and non-sitters based on a laboratory sitting assessment (see Procedure Section below): the final sample comprised 23 sitters (*M* = 29.29 weeks, *SD* = 0.77 weeks, range = 27.86–30.29 weeks, 13 boys and 10 girls) and 21 non-sitters (*M* = 28.34 weeks, *SD* = 1.17 weeks, range = 26.43–30 weeks, 9 boys and 12 girls). Infants were from predominantly middle-class families and were reported to be Caucasian (*n* = 38), African American (*n* = 1), Asian (*n* = 2), Multiracial (*n* = 1), or chose not to answer (*n* = 2). All infants were healthy and full-term, with no birth complications or vision problems. All mothers had graduated from high school, and 56.81% had completed at least a bachelor's degree. Four additional infants were tested but their data were not included in the final analyses due to experimenter error (*n* = 3) or parental interference (*n* = 1).

#### Procedure

Infants participated in three tasks. Infants first participated in the preferential looking task to assess sensitivity to symmetry as a cue to FG assignment. Immediately following the preferential looking task, infants participated in an in-laboratory sitting assessment. Finally, after a short break, infants were tested in a standard habituation task with unrelated stimuli.

#### Preferential looking

The stimuli, apparatus, and procedure for the test of infants' sensitivity to symmetry were the same as Experiment 1 with one exception: Experiment 2 contained only matched-motion trials. Infants were presented with 4 20-s, matched motion trials, the same number of matched motion trials as in Experiment 1. A second trained observer re-coded 25% of the infants off-line from a video record of the session. Mean inter-observer correlation for the duration of looking on each trial was high, *r* = 0.97, and the mean absolute difference between observers for the duration of looking was low, *M* = 0.45 s. Only data from the primary observer are reported here.

#### Sitting assessment

*One* of our main goals in Experiment 2 was to assess sitting in a more systematic way, and to validate the parent report measure used in Experiment 1. We assessed sitting using an adaptation of a sitting assessment developed by (Rochat, 1989). Infants were placed on a blanket on the ground in a sitting posture for 30 s. From the video records of this assessment, we classified infants as *sitters* only if they remained in a sitting posture the entire 30 s duration of the session and required no support from their arms or the experimenter. Note that although infants who are learning to sit may be able to sit unsupported for some period of time, *continuous* sitting frequently requires arm support (e.g., leaning on one or both arms) or support from the experimenter. Our interest is in *stable* sitting, and for this reason, infants who required support from their arms or the experimenter, as well as infants whose trunk folded onto their lap, or began to topple over, were classified as non-sitters. Two trained coders classified every session using frame-by-frame analysis. The agreement between these two coders was very high, 90%, and the remaining 10% were resolved between the two coders and constitute the final classifications. Importantly, agreement between the laboratory classification and parental report for sitters was 100%, thus parental report was used as a proxy for the lab assessment if infants were too fussy to complete the sitting assessment (*n* = 3), or in the case of experimenter error (*n* = 1).

It must be pointed out that although this sitting assessment is an improvement over the parental report used in Experiment 1, it is possible that we did not capture sitting differences as completely as we would have had we used a more standardized measure such as the Alberta Infant Motor Scale (AIMS). Our results may have been stronger if we had used such a measure, and therefore this is a consideration for future research.

#### Habituation

Infants were habituated (using a sliding-trial-block habituation criterion of 50% decrease in looking) to a single event in which a colorful novel object was manipulated and made some sound (e.g., it was rolled and it clicked). Trial durations were infant controlled; the stimulus remained visible for up to 35 s, or until the infant looked away for 1 s. The habituation phase ended when the infant habituated, or when they had completed 18 trials. Immediately following habituation, infants were tested on 3 novel stimuli, two that shared a single feature with the habituation stimulus (e.g., familiar sound or familiar action), and one that was completely novel. Our dependent measures from the habituation task were of processing speed (total looking time on the initial block of habituation trials, trials to habituation) and response to novelty (dishabituation to the completely novel event).

### Results

**Figure 4:**
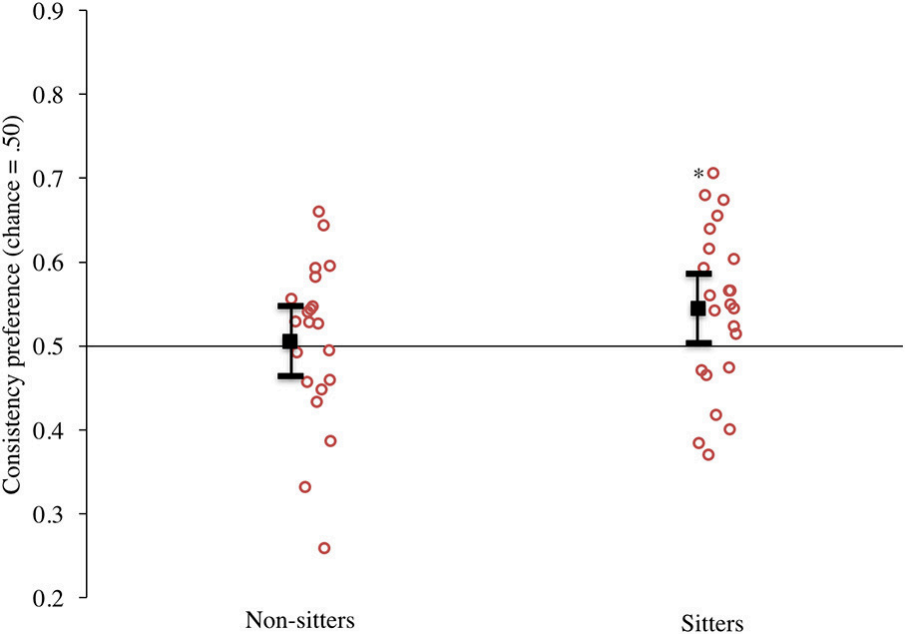
**Experiment 2 consistency preference scores by sitting status**. Circles represent individual infants, solid black square represents mean responding. (Note: ^*^denotes *p* < 0.05, error bars represent 95% confidence intervals).

Our primary analyses were those that evaluated the consistency preference scores for the sitters and non-sitters. The data are presented in Figure 4. Once again, the mean responding for the sitters (presented on the right) to these matched motion trials was >0.50, and most infants in this group had scores above this level. Comparing infants' responding to chance indeed confirmed that as in Experiment 1, sitters had a significant preference for the consistent event, *t*_(22)_ = 2.20, *p* = 0.04, *d* = 0.94. The non-sitters showed no clear preference; their mean responding was near 0.50, and individual infants' scores were divided above and below 0.50, *t*_(20)_ = 0.25, *p* = 0.81, *d* = 0.11. It is interesting to point out that this replication of the matched motion trials did not yield a tendency for non-sitters to exhibit a preference for the inconsistent events. Because of this, consistency preference scores between sitters and non-sitters did not differ significantly, *t*_(42)_ = −1.33, *p* = 0.19, *d* = −0.41.

#### Figure/ground segregation and habituation

A second goal of Experiment 2 was to determine whether sitters and non-sitters differ generally in other cognitive measures, as a possible explanation or the difference in FG segregation. First, to determine whether sitting infants were more cognitively advanced than non-sitting infants, we compared the performance of sitters and non-sitters on the habituation task. Thirty-nine of the 44 infants (21 sitters and 18 non-sitters) also contributed data to the habituation task. Sitters and non-sitters did not differ in duration of looking during the first habituation block, *t*_(37)_ = −1.88, *p* = 0.07, *d* = −0.62, the number of trials required to habituate, *t*_(37)_ = 0.81, *p* = 0.42 *d* = 0.27, or dishabituation to the completely novel event, *t*_(37)_ = 0.12, *p* = 0.91, *d* = 0.04. Of the 5 infants who failed to complete this task, 2 were sitters and 3 were non-sitters. Thus, sitting and non-sitting infants perform equivalently on a completely unrelated cognitive task suggesting that the two groups did not differ in general cognitive ability, at least as assessed by this habituation task.

We also conducted *t*-tests comparing infants' consistency preference scores as a function of performance on the habituation task. To accomplish this, we created median splits based on looking during the first habituation block, trials to criterion, and novelty preference (split at 0.5). These comparisons did not yield significant differences, all *p*s ≥ 0.15. In addition, comparisons of the average visual preference scores for each group to chance revealed that none of these groups (e.g., long lookers, short, lookers, fast habituators, slow habituators, high novelty preference, low novelty preference) had preference scores that differed from chance, all *p*s ≥ 0.07.

### Discussion

Experiment 2 replicated and extended the results of Experiment 1, by making use of an in-laboratory sitting assessment to provide increased precision and confidence in our classification of infant sitting ability. Importantly, the percent agreement between the in-laboratory sitting assessment used in Experiment 2 and parental report measure used in both Experiments 1 and 2 was 100%. In addition, Experiment 2 confirmed that sitters and non-sitters did not differ in an unrelated cognitive task, indicating that sitting infants are not simply more developmentally advanced than non-sitting infants. Rather, stable sitting appears to be related to emerging sensitivity to the FG assignment cue of symmetry, suggesting the importance of motor development to the development of selective attention, object perception, and FG segregation.

## General discussion

In two experiments, we demonstrated that at 6.5 months of age, infants' sensitivity to the cue of symmetry for FG segregation was related to self-sitting abilities. In each experiment, infants who could sit independently showed sensitivity to symmetry as a cue to FG segregation whereas infants who could not yet sit independently did not show such sensitivity. These results contribute to our understanding of the development of infant visual perception in three important ways. First, although previous research reveals some of infants' developing abilities to segregate objects in complex visual arrays (von Hofsten and Spelke, 1985; Craton and Yonas, 1988; Needham, 2001; Yonas et al., 2002; Kavsek et al., 2009), this study is the first to demonstrate infants' sensitivity to the Gestalt cue of symmetry for FG assignment.

Second, these findings provide insight into how this sensitivity develops. Like pictorial depth cues, FG cues such as size, convexity, and symmetry reflect the environmental regularities and non-accidental properties of objects. For example, a symmetric region is more likely to arise from a symmetric foreground object than from a symmetric background region formed by two foreground objects that happen to have the same contour. Infants may learn such regularities with experience. Indeed, infants are particularly good at detecting and using statistical regularities such as these to learn about the auditory and visual world (e.g., Aslin et al., 1998; Kirkham et al., 2002), and use of these learned regularities persist into adulthood (see Aslin and Newport, 2012), including regularities that organize or group visual information (Vickery and Jiang, 2009; Zhao et al., 2014). We observed that sensitivity to the regularities that facilitate FG assignment emerges between 6 and 7 months, the same age range infants become sensitive to several pictorial depth cues (Yonas et al., 2002). Therefore, between 5 and 7 months infants appear to become sensitive to some of the regularities that help them arrange in depth the objects in complex visual arrays.

Finally, these findings suggest that the ability to sit unsupported is a potential mechanism for the emergence of sensitivity to symmetry as a cue to FG assignment. Research has established that sensitivity to the unique properties of vertical symmetry emerges around 4 months of age (Bornstein et al., 1981; Fisher et al., 1981), however the use of symmetry as a reliable FG assignment cue may not develop until substantially later. We propose that once infants begin to sit independently, infants learn that symmetry is a regular characteristic of *objects*, not backgrounds. Experiments 1 and 2 constitute the first steps toward testing this hypothesis, and the results clearly show that sitting infants, and not those infants that cannot yet sit, preferred to look at the stimulus events consistent with the symmetrical figure.

It should be noted that it is impossible to know exactly why infants demonstrate a preference for the consistent displays. One possibility is that infants simply prefer to look at displays that contain non-deforming symmetry, and that they are not necessarily using symmetry as a FG segregation cue. This is unlikely for three reasons. First, we know displays that contain occlusion and motion like ours produce a robust and likely automatic percept of figure and ground (Kellman and Spelke, 1983; Craton and Yonas, 1988; Cohen and Cashon, 2001; Johnson et al., 2003; Bremner et al., 2015)—each composite contains a single motion-defined figure, and a ground. Thus, it is impossible to interpret a preference for consistent displays independent of the motion-defined FG segregation. Second, despite early sensitivity and even preference for vertical symmetry (Bornstein et al., 1981; Fisher et al., 1981; Bornstein and Krinsky, 1985) sensitivity to symmetry embedded in moving occlusion displays emerges only after infants learn to sit. We and others (Soska et al., 2010; Baumgartner and Oakes, 2013; Corbetta et al., 2014) have suggested that visual and/or motor experiences, such as those that accompany independent sitting, result in increased attention toward plausible objects, and infants have learned that symmetry is a reliable indicator of objectness. Finally, even if infants were able to ignore the motion-defined FG segregation cues and simply preferred to look at non-deforming symmetry, we would expect to find a preference for the consistent displays for both matched and unmatched motion conditions—but we do not.

Why is sitting related to sensitivity to symmetry as a FG assignment cue? One possibility is that the postural control that accompanies stable sitting may allow the infant to demonstrate visual preferences in the task used here. That is, because sitting infants have better postural control, they may be better able to look back and forth between two visually presented stimuli and show a systematic preference for one type of stimulus over another. However, because visual preferences for both static (Fantz, 1958) and dynamic (Ross-Sheehy et al., 2003) stimuli have been revealed in the preferential looking procedure in infants 4 months and younger, this is an unlikely reason for the observed differences between sitters and non-sitters.

A second, more likely, possibility is that stable sitting has consequences for infants' manual and visual exploration of the world, and that this new means of exploration provides the opportunity to discover the regularities that define object boundaries. Infants who can sit unsupported have acquired the postural control required to extend the arms away from the body and reach for the objects that surround them. Clearly the ability to obtain objects from a cluttered visual array provides infants access to information about the regularities that specify object boundaries. In addition, this increased motor experience may help tune their developing perception/action system, allowing them to interact with objects more efficiently. For example, 7-month-old infants have been shown to orient their grasp prior to grasping an object, whereas 5-month-old infants do not, suggesting the important role of prior reaching experience in motor planning and perception for action (McCarty et al., 2001; Witherington, 2005). In addition, selective visual attention to the graspable part of an object has been shown to increase with increased reaching experience (Corbetta et al., 2014). It is possible that infants learn to use object information specified visually through the process of visually identifying to-be-grasped objects, grasping objects, haptically exploring objects, and using tactile feedback to readjust their grasp. Similarly, as the child acquires experience reaching for and successfully grasping objects such as a rattle or a teddy bear, the more the child will learn that graspable objects share some perceptual commonalities, such as symmetry.

Finally, sitting infants likely spend more time each day looking *vertically* at the world; a perspective that may increase infants' detection of symmetry in everyday objects such as faces, bottles, and furniture (Zhao et al., 2014), and early visual experience appears to be critical (Cattaneo et al., 2010, 2013). Recent work suggests that vertical symmetry perception happens early in visual processing, may lead to enhanced or automatic “object-based attention,” and results in qualitatively different patterns of neural activation than other forms of object perception (Apthorp and Bell, 2015; Bertamini et al., 2015; Bona et al., 2015; see also Hecht et al., 2016). Thus, it is possible that stable sitting enhances sensitivity to the FG cue of symmetry either through increased visual experience (i.e., statistical learning), increased haptic and motor experience, by providing a more ideal visual perspective to automatically detect vertical symmetry in the environment, or some combination of all three. Future work should be aimed at further refining the relationship between stable sitting and the use of symmetry in FG assignment.

The relationship we propose between unsupported sitting and sensitivity to symmetry as a cue to FG assignment is similar to that observed between self-produced locomotion and the emergence of heights wariness. That is, despite early perceptual sensitivity to visual features such as depth, only increased locomotor experience results in categorically different perception/action plans—avoiding rather than plunging in to unsafe gaps and drop-offs (Campos et al., 1992; Adolph et al., 2014). Here too we suggest that despite early perceptual sensitivity to the statistical redundancies of vertical symmetry (Bornstein et al., 1981; Fisher et al., 1981), only with sitting and subsequent changes in reaching and/or visual experience do infants come to rely on the use of symmetry as a FG assignment cue. Though future work could benefit from a more precise assessment of both of sitting and reaching, these results represent a significant first step toward understanding the complex relations between sitting, motor and visual experience, and visual perception.

In summary, these findings add to our understanding of the development of visual perception in infancy. We have demonstrated that young infants are sensitive to Gestalt cues to FG assignment and that this sensitivity emerges at approximately the same time as sensitivity to pictorial depth cues. These results are compatible with the view that infants' acquisition of these FG cues is a function of their ability to detect and learn statistical regularities and that motor achievements create new opportunities to learn those regularities.

## Author contributions

SR, SP, SV, and LO worked together to develop the experiments and techniques presented in this manuscript. SR and SP created and animated the figure-ground stimuli, SP, and LO developed the habituation procedures, and SP, developed the sitting assessments and coding schemes. Data were collected by SR and SP. SR, SP, and LO worked together on the analyses and interpretation of the results, and SR wrote the manuscript with help from SP, SV, and LO Funds, resources, and oversight were provided by LO and SV, with some addition funding provided by SR.

### Conflict of interest statement

The authors declare that the research was conducted in the absence of any commercial or financial relationships that could be construed as a potential conflict of interest.

## Footnotes

^1^Although this comparison appears to be marginal (*p* = 0.06), recall that we used a Bonferroni's correction to adjust for multiple comparisons, and our more conservative criterion for significance was *p* ≤ 0.0125. Moreover, any difference between non-sitters for the two types of trials is due to many infants in this group exhibiting a preference that was *less* than chance. However, it is clear from the distribution of individual scores presented in Figure 3 that these infants did not exhibit a robust and systematic preference for the inconsistent event. The replication in Experiment 2 will address the possibility that non-sitting infants actually prefer the inconsistent event.
